# The Predictive Role of the Histopathological Scoring System in Adipose Tumors—Lipoma, Atypical Lipomatous Tumor, and Liposarcoma

**DOI:** 10.3390/diagnostics13243606

**Published:** 2023-12-05

**Authors:** Mariana Deacu, Madalina Bosoteanu, Manuela Enciu, Georgeta Camelia Cozaru, Oana Cojocaru, Gabriela Izabela Baltatescu, Anca Antonela Nicolau, Cristian Ionut Orasanu, Bogdan Marian Caraban, Raluca Ioana Voda

**Affiliations:** 1 Clinical Service of Pathology, Department of Pathology, “Sf. Apostol Andrei” Emergency County Hospital, 900591 Constanta, Romania; deacu_mariana@yahoo.com (M.D.); mbosoteanu@yahoo.com (M.B.); iftimemanuela@yahoo.com (M.E.); cojocaru_oana@yahoo.com (O.C.); gabrielabaltatescu@yahoo.com (G.I.B.); ancanicolau@rocketmail.com (A.A.N.); raluca.v1694@yahoo.ro (R.I.V.); 2 Faculty of Medicine, “Ovidius” University of Constanta, 900470 Constanta, Romania; bcaraban@yahoo.com; 3 Center for Research and Development of the Morphological and Genetic Studies of Malignant Pathology (CEDMOG), “Ovidius” University of Constanta, 900591 Constanta, Romania; drcozaru@yahoo.com; 4 Clinical Service of Pathology, Department of Genetics, “Sf. Apostol Andrei” Emergency County Hospital, 900591 Constanta, Romania; 5 Clinical Department of Plastic Surgery, Microsurgery—Reconstructive, “Sf. Apostol Andrei” Emergency County Hospital, 900591 Constanta, Romania

**Keywords:** atypical lipomatous tumor, CDK4, histopathological score, lipoma, liposarcoma, MDM2, microvascular density

## Abstract

Lipomatous tumors are the most frequent soft tissue neoplasms. Sometimes their differential diagnosis is difficult to perform only by microscopic analysis. This study aims to create a histopathological scoring system and highlight the impact of intratumoral microvascular density. This study was conducted over 10 years. We analyzed the main pathogenic pathways (MDM2 and CDK4), as well as the tumor microvascularization (CD31 and CD34) by immunohistochemical tests. We also analyzed the status of the MDM2 gene by CISH. These data, together with the clinical and histopathological information, were statistically analyzed by appropriate tests. We identified 112 eligible cases, with most of the patients being in their sixth decade of life, with a slight predominance of the female sex. We found important associations like tumor location linked to nuclear pleomorphism severity and microvascularization density correlated with atypia severity. Also, we observed that a maximum diameter of a tumor of at least 69 mm is associated with the presence of tumor necrosis. The score designed in this study shows an increased sensitivity and specificity for the diagnosis of lipomas (100%, respectively, 97%), atypical lipomatous tumors (93.8%, respectively, 82.3%), and liposarcomas (100%, respectively, 90.5%). This present study enhances the present data by bringing to attention the histopathological score with a role in differential diagnosis, as well as in the prediction of immunohistochemical and genetic tests. Also, we highlighted the importance of microvascular density, especially in the diagnosis of liposarcomas.

## 1. Introduction

The most frequent type of soft tissue tumors are neoplasms originating from adipose tissue. Lipomas are the most common and were first described in 1856 by Sir James Paget. Their incidence is underreported, with most of them being asymptomatic. They can be located superficially (most often) but also in the deep soft tissue [1].

Atypical lipomatous tumor/well-differentiated liposarcoma (ALT/WDLS) represents a spectrum of malignant tumors of adipose tissue, locally invasive, but without metastatic characteristics. The two terms used can be differentiated based on their location and resectability. Thus, for lesions located on the limbs, only the term atypical lipomatous tumor can be used because it has a higher rate of resectability. In retroperitoneal locations, the term well-differentiated liposarcoma can be used because it has a higher rate of recurrence and dedifferentiation [2]. They are usually located deep (at the proximal level of the extremities, retroperitoneal, and mediastinal) and are less often superficial (subcutaneous) [2,3].

Liposarcomas are the most common malignant tumors of soft tissue (15–20%). If the well-differentiated liposarcoma is of low grade, the other subtypes (dedifferentiated, myxoid, and pleomorphic) are of high grade, with a high capacity for recurrence and metastasis [4].

The technique of fluorescence in situ hybridization (FISH) has proven to be extremely valuable in assessing the MDM2 status in cases where distinguishing between ALT/WDLS and benign lipomatous neoplasms is challenging based solely on morphological features [5]. The genomic profile of lipomatous tumors has been successfully identified through the effective utilization of cytogenetic and molecular analysis. For example, ALT/WDLS and dedifferentiated liposarcoma are types of tumors known for their distinct genetic characteristics, particularly the amplification of the mouse double minute 2 (MDM2) oncogene on the 12q13-15 region [6].

However, FISH necessitates the use of specialized equipment for observing fluorescence signals and requires interpretation along with corresponding light microscopic sections. Lately, there have been exciting advancements in bright-field in situ hybridization techniques, particularly chromogenic in situ hybridization (CISH). These techniques have proven to be invaluable in determining the amplification status of MDM2. By supplementing histopathology, they have become an essential tool in differentiating liposarcoma from other types of soft tissue neoplasms [5].

The objective of this study is to assess the main clinical, morphological, immunohistochemical, and genetic features of lipomatous tumors diagnosed over 10 years. We aim to develop a scoring system that will greatly benefit both pathologists and clinicians by enabling them to accurately diagnose and effectively manage these tumors. In addition, a significant advantage of this study is the evaluation of the intratumoral vascularization in the cases of the study group. This aspect holds significant value in the progression of tumor formation and has not been thoroughly explored in these specific types of tumors. Its main objective is to identify correlations between microvascular density and pathomorphological factors.

## 2. Materials and Methods

**Figure 1:**
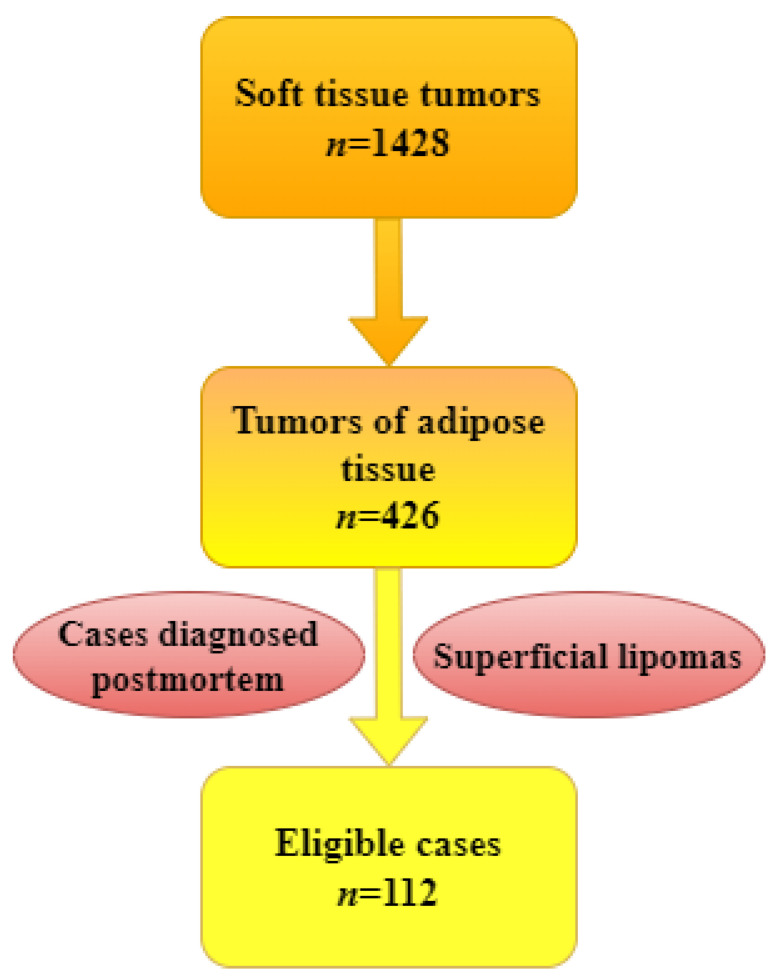
Flow chart with the inclusion and exclusion criteria of the selected cases.

We conducted a retrospective study for a period of ten years (2013–2022) of patients diagnosed with adipose tissue tumors hospitalized at the Constanta County Emergency Clinical Hospital, Romania. The data were extracted from the hospital’s archive and electronic databases. The inclusion criteria in this study were represented by benign and malignant adipose tissue tumors diagnosis. Exclusion criteria included superficial localization of benign adipous tumors that did not require a differential diagnosis with malignant tumors, as well as cases diagnosed post-mortem (Figure 1).

The clinical information (age, gender, localization, depth, and presence or absence of pain) of the patients came from the hospitalization form.

The surgical excision specimens were macroscopically described (with emphasis on maximum diameter) and prepared according to international protocols, up to the stage of microscopic slides in the usual staining (Hematoxylin–Eosin) within the Clinical Anatomic Pathology Service of Constanta. The histopathological evaluation was carried out by two pathologists, and the old cases were re-evaluated according to the latest criteria of the World Health Organization (WHO 2020), regarding soft tissue pathology. The histopathological examination evaluated: cellular pleomorphism (low, moderate, or high), nuclear atypia, karyomegaly, presence of lipoblasts (absent, rare, or frequent), necrosis (absent, <50% or >50%), and the number of mitoses per 10 HPF.

The immunohistochemical examinations were conducted at the Center for Research and Development of The Morphological and Genetic Studies of Malignant Pathology (CEDMOG), University Ovidius Constanta, with evaluation performed by two separate pathologists. The formalin-fixed paraffin-embedded samples were sectioned at a thickness of 4 μm and prepared following the established protocol provided by Master Diagnostica (Sevilla, Spain). To ensure accuracy, positive control slides were included in each execution.

**Figure 2:**
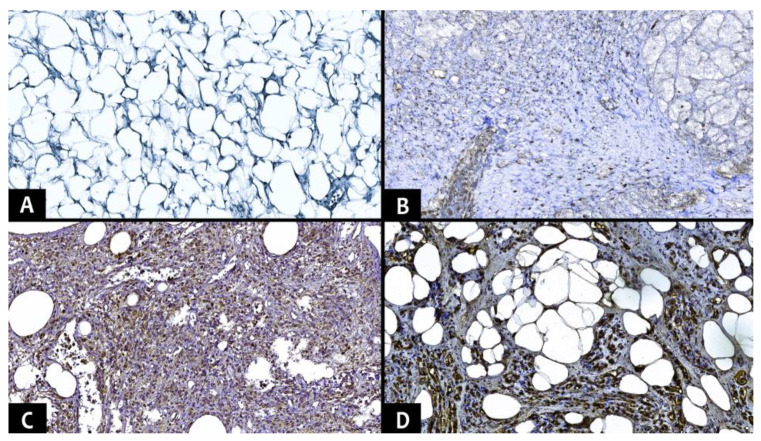
Different immunohistochemical aspects of the MDM2 marker (Ob. ×100). (**A**). Lipoma with negative expression. (**B**). Dedifferentiated liposarcoma with positive immunoexpression <25%. (**C**). Pleomorphic liposarcoma with epithelioid morphology with positive immunoexpression 25–50%. (**D**). Atypical lipomatous tumor mixed subtype with positive immunoexpression >50%.

**Figure 3:**
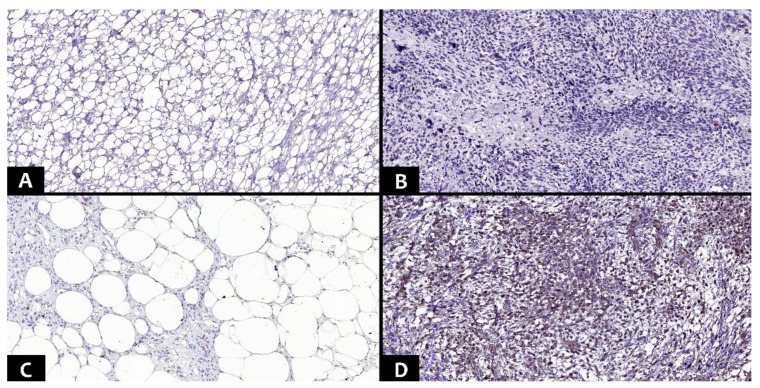
Different immunohistochemical aspects of the CDK4 marker (Ob. ×100). (**A**). Lipoma with negative expression. (**B**). Dedifferentiated liposarcoma with positive immunoexpression <25%. (**C**). Atypical lipomatous tumor, lipoma-like subtype with positive immunoexpression 25–50%. (**D**). Myxoid liposarcoma with positive immunoexpression > 50%.

Immunohistochemical tests used the markers MDM2 (IF2, ready-to-use, HIER-DAB method), CDK4 (DCS-35, ready-to-use, HIER-DAB method), CD34 (QB-END/10, ready-to-use, HIER-DAB method), and CD31/PECAM-1 (EP78, ready-to-use, HIER-DAB method). Hematoxylin–Eosin was used for the counter-staining procedure. Nuclear markers (MDM2 and CDK4) were quantified as the percentage of positive nuclei on 10 high power fields (HPF), in at least 1000 nuclei. The immunoreaction was classified into four distinct levels: negative, <25%, 25–50%, and >50% (Figure 2 and Figure 3).

**Figure 4:**
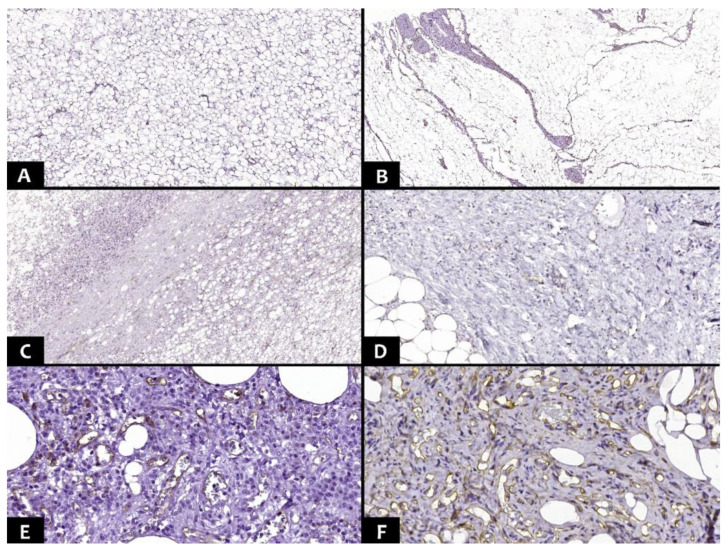
(**A**). CD31+ expression at the level of the endothelium in the lipoma (1 mm^2^/Ob. ×25). (**B**). CD34+ expression at the level of the endothelium in the lipoma (1 mm^2^/Ob. ×25). (**C**). CD31+ expression at the level of the endothelium in the atypical lipomatous tumor (1 mm^2^/Ob. ×25). (**D**). CD34+ expression at the level of the endothelium in the atypical lipomatous tumor (Ob. ×100). (**E**). CD31+ expression at the level of the endothelium in the liposarcoma (×200). (**F**). CD34+ expression at the level of the endothelium in the liposarcoma (×200).

Membrane markers (CD34 and CD31) were evaluated as positive at the level of endothelial cells (Figure 4). Thus, for the evaluation of the microvascular density, the slides were scanned with a TissueScope LE120 Slide Scanner (Huron Digital Pathology, ON, Canada), capturing images made up of 10 hotspot areas of microvascularization (for each marker). The total number of vessels identified by two pathologists was divided by 10 (for each marker separately), and the average of the results represented the mean number of vessels per 1 mm^2^.

**Figure 5:**
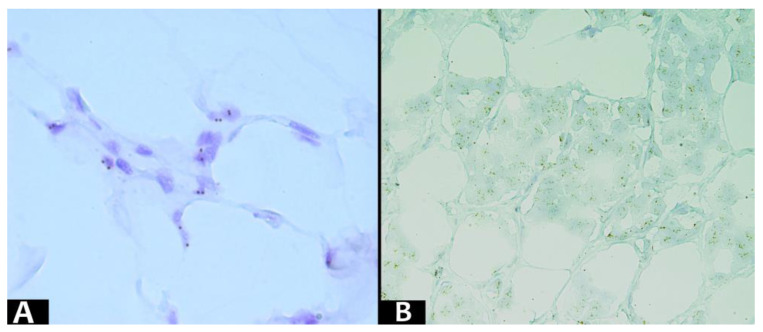
Representative photomicrograph of CISH analysis of MDM2 gene (brown signals) in a paraffine section, showing: (**A**) normal status of MDM2 gene and (**B**) amplification of MDM2 gene.

We utilized the ZytoDot SPEC MDM2 Probe (Bremerhaven, Germany), a Digoxigenin-labeled probe specially designed for detecting MDM2 gene amplification at the 12q15 region, through Chromogenic in situ hybridization (CISH). The sections were prepared by cutting formalin-fixed paraffin-embedded samples into 3 μm-thick slices. Then, the tissue slides were processed following the manufacturers’ guidelines, encompassing pretreatment, denaturation, hybridization, and post-hybridization procedures. The CISH signals were observed using a high-powered light microscope—Zeiss AxioScope A1 (Zeiss Gmbh, Jena, Germany)—and a 100× objective lens with oil immersion. The hybridization signals of Digoxigenin-labeled polynucleotides are visible as distinct dots, ranging in color from brown to dark brown. For accurate interpretation, a minimum of 20 cells were counted and analyzed. Non-neoplastic cells must exhibit two clearly identifiable dot-shaped signals within each nucleus. Nuclei displaying amplification of the MDM2 gene locus or polysomy of chromosome 12 will exhibit multiple dots or large signal clusters (Figure 5).

The statistical analysis of the data was conducted using SPSS Statistics Version 26 (IBM Corporation, New York, NY, USA). We utilized indicators of central tendency and variability. For the analysis of univariate data, we utilized Fisher’s exact test for categorical data and both the Mann–Whitney U Test and the Kruskal–Wallis H test for continuous variables, as appropriate. To measure the association of the data, we used the Pearson correlation coefficient, and for the prediction of the response between variables, the Pearson regression. Receiver operating characteristic (ROC) and area under the curve (AUC) were used to establish the accuracy of the parameters. The sensitivity and specificity of the parameters are the optimal cutoff point as the value that maximizes the area under the ROC curve. The results reached statistical significance with a *p*-value of less than 0.05.

Every patient had signed an informed consent upon admission. We have obtained an ethical opinion from the local ethics commission (Ethics Commission of the Constanta County Emergency Hospital).

## 3. Results

### 3.1. Clinical Aspects

After applying the inclusion and exclusion criteria, 112 cases were detected. The majority were lipomas (70.53%), followed by liposarcomas (15.18%) and ALT/WDLS (14.29%) (Table 1).

Most of the patients were in the sixth decade of life (26.13%). The average age was 53.35 years (3–86 years), with a slight female predominance (51.79%). Locations in the upper trunk (26.79%), lower limbs (20.54%), and retroperitoneal (15.18%) were the most common. Of the malignant cases, 15 (45.45%) were superficially located. Approximately half of the patients complained of pain at presentation (50.89%). Most of the lesions had a diameter below 10 cm (85.71%), with the average being 64.45 mm (11–290 mm) (Table 2).

We noticed a difference between diagnoses and the presence of pain; this was associated with cases of malignancy (*p* = 0.010).

### 3.2. Histopathological Aspects

A statistically significant association was observed between cellular pleomorphism and biological potential; thus, low pleomorphism was associated with lipoma and ALT/WDLS, while moderate or increased pleomorphism was associated with liposarcoma (*p* < 0.001). The presence of rare lipoblasts was correlated with the diagnosis of ALT/WDLS, while an increased number of lipoblasts was associated with liposarcoma (*p* < 0.001). Liposarcoma was associated with a percentage of more than 50% of tumor necrosis, while ALT/WDLS with a percentage of less than 50% (*p* < 0.001). Malignant lesions were correlated with the presence of intratumoral fibrous proliferation (*p* < 0.001) (Table 3).

Following the associations made, MDM2 immunopositivity in the case of liposarcoma is over 25% of the nuclei, while in the case of ALT/WDLS, it is over 50% of the nuclei (*p* < 0.001). Similarly, we observed that CDK4 immunopositivity, in malignant cases (ALT/WDLS and liposarcomas), must be over 25% of the nuclei (*p* < 0.001) (Table 3).

We noticed that older age was associated with the presence of nuclear atypia (*p* = 0.042) and an increased number of lipoblasts (*p* = 0.034). Males accused pain more frequently than females (*p* = 0.015). Also, subcutaneous locations were correlated with the presence of pain (*p* = 0.024). The presence of nuclear atypia, karyomegaly, and increased cellular pleomorphism (*p* = 0.008, *p* = 0.021, respectively, *p* = 0.002) was associated with the presence of pain, while the foci of necrosis did not present associations with pain (*p* = 0.526).

As expected, superficial lesions were correlated with the presence of nuclear atypia (*p* < 0.001), karyomegaly (*p* < 0.001), and fibrous proliferation (*p* = 0.005), while those with deep localization were associated with cellular pleomorphism moderate to high (*p* = 0.020) and with an increased microvascular density (*p* < 0.001).

The presence of karyomegaly and fibrous proliferation was associated with an increased maximum diameter (*p* = 0.014, respectively, *p* < 0.001). In the case of the present fibrous proliferation, we identified a cut-off of the maximum diameter of ≥51 mm, having a sensitivity of 75% and a specificity of 60.5% (AUC 0.739, *p* < 0.001). The larger the maximum diameter of the tumor, the higher the number of mitoses and the microvascular density (*p* = 0.002, respectively, *p* = 0.008). The presence of nuclear atypia was associated with the presence of intratumoral fibrous proliferation, as well as with the presence of necrosis (*p* < 0.001, respectively, *p* < 0.001). A strong association could be highlighted between the presence of cellular atypia and microvascular density. Thus, a cut-off of ≥16.25 vessels/mm^2^ had a sensitivity of 100% and a specificity of 92.9% (AUC = 0.989, *p* < 0.001), in the case of the presence of cellular atypia.

Karyomegaly of tumor cells was associated with the presence of fibrous proliferation and necrosis (*p* < 0.001, respectively, *p* < 0.001). Likewise, moderate and increased cellular pleomorphism was related to the presence of intratumoral fibrocytic proliferation and necrosis (*p* = 0.035, respectively, *p* < 0.001).

The presence of intratumoral fibrosis was associated with the presence of lipoblasts and necrosis (*p* < 0.001, respectively, *p* = 0.017). It was observed, thus, a statistically significant association between the presence of fibrosis and a percentage of over 25% CDK4 immunopositivity.

A percentage below 50% of tumor necrosis was correlated with the presence of rare lipoblasts, while a percentage above 50% was associated with the presence of numerous lipoblasts (*p* < 0.001). Also, an increased number of lipoblasts confers a mitotic activity ≥ 3.5 mitoses/HPF. This threshold has a sensitivity of 91.7% and a specificity of 94% (AUC = 0.977, *p* < 0.001). A number of ≥4.5 mitoses/HPF are predictors for a percentage of over 50% of necrosis, with a sensitivity of 100% and a specificity of 92.5% (AUC = 0.972, *p* < 0.001). The same aspect was also found in the case of lesions of ≥69 mm but with lower sensitivity and specificity—83.3% and 68.9%, respectively (AUC = 0.779, *p* = 0.022).

In malignant cases, there is a statistically significant correspondence between MDM2 and CDK4 immunomarkers. Thus, a CDK4 immunopositivity of over 50% correlates with an MDM2 immunopositivity of over 25%. From a cytogenetic point of view, only an MDM2 of over 50% corroborates with gene amplification but without statistical significance (*p* = 0.282). On the other hand, a CDK4 of over 25% is associated with the amplification of the MDM2 gene (*p* = 0.012). A cut-off of ≥38.75 vessels/mm2 is predictive for liposarcoma with 94.1% sensitivity and 100% specificity (AUC = 0.999, *p* < 0.001). Also, for liposarcoma, the mitotic activity of ≥4.5 mitoses/HPF shows a sensitivity of 82.4% and a specificity of 100% (AUC = 0.974, *p* < 0.001).

### 3.3. Histopathological Score

To develop a predictability score, the clinical and histopathological aspects were entered into a multivariable regression (Table 4).

After this analysis, we noticed that only the variables: nuclear atypia, cellular pleomorphism, fibrous proliferation, lipoblasts, and mitosis have statistical significance (*p* < 0.005). To these data, the ROC curve is added, which indicates a sensitivity of 82.4% and a specificity of 100% for ≥4.5 mitoses in the case of liposarcoma (AUC = 0.996, *p* < 0.001) and a sensitivity of 81.30% and a specificity of 82.3% for ≥1.5 mitoses in the case of ALT/WDLS (AUC = 0.814, *p* < 0.001). These data were quantified as follows in Table 5.

**Figure 6:**
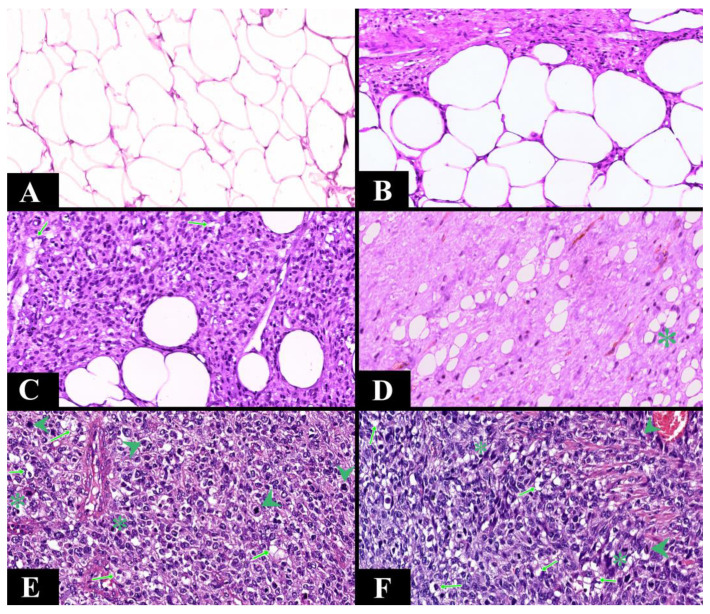
Representative photomicrographs of adipose tumor cases at Ob. ×200 in Hematoxylin–Eosin staining. (**A**). Lipoma—adipocytes without nuclear atypia (0 points), with low cellular pleomorphism (0 points), not accompanied by fibrous proliferation (0 points), absent lipoblasts (0 points), and without mitotic activity (0 points)—score 0. (**B**). Lipoma—adipocytes without nuclear atypia (0 points), with low cellular pleomorphism (0 points), accompanied by fibrous proliferation (1 point), absent lipoblasts (0 points), and without mitotic activity (0 points)—score 1. (**C**). ALT/WDLS—adipocytes without nuclear atypia (0 points), with low cellular pleomorphism (0 points), accompanied by fibrous proliferation (1 point), with rare lipoblasts (1 point), and without mitotic activity (0 points)—score 2. (**D**). ALT/WDLS—adipocytes with nuclear atypia (1 point), with moderate cellular pleomorphism (1 point), accompanied by fibrous proliferation (1 point), absent lipoblasts (0 points), and no mitotic activity (0 points)—score 3. (**E**). Liposarcoma—adipocytes with nuclear atypia (1 point), with moderate cellular pleomorphism (1 point), not accompanied by fibrous proliferation (0 points), frequent lipoblasts (2 points), and >4 mitoses/HPF (2)—score 6. (**F**). Liposarcoma—adipocytes with nuclear atypia (1 point), with increased cellular pleomorphism (2 points), accompanied by fibrous proliferation (1 point), frequent lipoblasts (2 points), and 2–4 mitoses/HPF (1 point)—score 7. Legend: asterisk (*)—nuclear atypia, long arrow (→)—lipoblasts, and arrowhead (►)—mitoses.

On the sum of the points, we used the ROC curve and identified that a practical score of 0–1 is predictive for lipoma, a score of 2–3 for ALT/WDLS, and a score of four and above for liposarcoma (Table 6, Figure 6).

This score can also be used in the prediction of immunohistochemical markers (MDM2 and CDK4) and the status of the MDM2 gene (Table 7).

## 4. Discussion

Adipose tissue neoplasms are the most common soft tissue tumors. They have a mesenchymal origin and are usually located on the trunk and limbs. Also, in the case of our study, most cases were located at these levels. These tumors are characterized by histological heterogeneity [7]. The etiology of these tumors is unknown. However, genetic predisposing factors and extrinsic factors have been identified, such as ionizing radiation, exposure to dioxins, chlorophenol, and some viruses (HIV) [8].

Lipoma and its subcategories represent over half of soft tissue tumors, with a prevalence of 21/100 people per year [7]. Other authors claim that the prevalence of lipomas is 1 in 100 people globally. In the case of multiple lesions, the prevalence increases to 5% [9]. They usually appear in the 5th–7th decades of life, are rare in the pediatric population, and have been associated with obesity [10]. In the case of our study, we noticed that the majority of lesions were lipomas, with a percentage of 70.53%, and the average age at the time of diagnosis of the lipoma cases analyzed was 51.81 (3–77 years). Most of the diagnosed patients were female (54.43%).

Atypical lipomatous tumor/well-differentiated liposarcoma/dedifferentiated liposarcoma (ALT/WDLS/DDL) is a common type of liposarcoma in late adulthood. However, there are reports of pediatric ALT/WDLS/DDL, although these are extremely rare [11]. ALT/WDLS is a locally aggressive mesenchymal neoplasm without metastatic potential. It accounts for 40–45% of all liposarcomas. Males and females are equally affected, with a peak incidence occurring between the fourth and fifth decades of life [12].

In this research, we observed that 14.29% of the tumors were ALT. The age at the time of diagnosis varied between 30 and 68 years (average—53.13) and 62.5% of the diagnosed patients were male.

According to the American Cancer Society, liposarcoma is the most common soft tissue sarcoma worldwide. No other predilection for liposarcoma is known. There is no significant association with race or sex. A slight male predominance has been reported in some studies [13]. It comprises up to 12.8% of all malignant soft tissue tumors and accounts for nearly 20% percent of adult mesenchymal tumors [14,15]. The etiology and risk factors of liposarcoma are unknown. According to CNPCR and SEER data, there was an increase in incidence among men until age 75–84 years, with peak rates of 4.36 and 4.95 cases per 100,000 person-years. Liposarcoma rates in women peaked at 75–84 years with the following estimates: 1.89 and 1.97 cases per 100,000 person-years in CNPCR and SEER data [15].

In this study, 15.18% of the cases were liposarcomas. The average age at the time of diagnosis was 61.19 (43–86 years). Unlike previous studies that reported a male predilection for these tumors, in the case of our research, we observed a slight predominance in the female sex (52.94%).

Lipomas appear as painless lesions, but large ones can cause compression of nerve structures with the appearance of pain or discomfort. Instead, ALT/WDLS presents itself as deeply localized, painless lesions, especially at the retroperitoneal level. In the case of this localization, the clinical manifestations begin when the tumor exceeds 20 cm. Dedifferentiated liposarcoma appears as a painless lesion, which can be discovered incidentally, and myxoid as a large, painless lesion. The pleomorphic variant is a rapidly growing lesion [10]. 

In this study, we identified pain as the main clinical manifestation that correlates significantly with the diagnosis of malignant tumors (*p* = 0.010). The presence of pain was more often found in cases of ALT and liposarcoma, correlating with the malignant potential. This aspect can be attributed to the selection of benign lesions with deep localization.

By evaluating CT or MRI, lipomas have typical features: lesions delimited by a discrete capsule, with characteristics similar to subcutaneous adipose tissue. At MRI, these tumors have a homogeneous appearance and isointense with the subcutaneous adipose tissue on all pulse sequences. In both imaging methods, thin internal striations determined by muscle fibers, blood vessels, or fibrous septa can still be observed, but these do not measure more than 2 mm and do not present any significant internal accentuation [10]. Most of the patients included in this study had one of these investigations during hospitalization so the diagnosis of adipose tissue tumors was made. 

MRI aspects indicating an atypical lipomatous tumor are a large lesional volume, atypical septation, the thickness of septa over 1.3 mm, a discrepancy between intratumoral Short-TI Inversion Recovery (STIR) without a perilesional fluid signal, contrast enhancement, and apparent diffusion coefficient alterations. Thus, tumor sphericity is much more significant for ALT. Aspects that strongly correlate with atypical lipomatous differentiation are a low fraction of adipose tissue, higher cellularity, and a higher content of fibrous tissue. Strongly indicative of ALT is also the nodular contrast pattern or the entire heterogeneous lesion [16]. The sensitivity and specificity of the MRI detection of ALT varies between 90.1 and 90.9%, respectively, and 37 and 51.5% [17,18].

Signs of malignant biological potential in the CT examination of soft tissue sarcomas are represented by irregular edges, infiltration of adjacent organs, calcifications, necrosis, and hypervascularization [19].

Imaging shows that ALT/WDLS is predominantly composed of at least 75% adipose tissue. The appearance is similar in the case of dedifferentiated liposarcoma; rarely, focal juxtapositions of nonlipomatous masses can be observed at its level. Nonadipose areas show a decrease in signal in T1-weighted images and a variable increase in signal in T2-weighted or fluid-sensitive images, as well as an increase in attenuation on CT examination. Hemorrhage and necrosis can be observed in high-grade dedifferentiated components [20].

Asano Y et al. proposed an evaluation score to differentiate lipoma from ALT/WDLS. This score includes imaging parameters obtained from the MRI examination (tumor size, location, thickness of intralesional septa, depth of lesion location, and accentuation of septa or nodular lesion) and a microscopic element (the presence of lipoblasts). The authors quantified the elements with points from 0 to 5. A score greater than nine points is highly suggestive of ALT/WDLS. Also, these researchers evaluated the sensitivity and specificity of the score both in the group used to develop the score and in the validation group. The sensitivity was 87.6% in the first group and 80% in the validation group, and the specificity was 91.1% in the development group and 96.7% in the validation group [21].

Nagano et al. have developed a score for differentiating lipoma from ALT that includes the diameter of the lesion, the depth of the location, the presence of intratumoral septa, and the post-contrast substance administration accentuation examined by MRI. Each parameter corresponds to a score from 0 to 2. All lipomas they evaluated using this score had a low final score, with an average of 1.7 points, while ALT had much higher values, with an average of 5.1 points. The authors observed that this score had a sensitivity of 100% and a specificity of 77% [22].

Cheng et al. proposed a lipomatous tumor evaluation score using a logarithmic formula based on age, sex, lesion location, septal thickness, and post-contrast contrast enhancement or fat component < 75% [23].

In this study, we created a score based on the histopathological characteristics: the severity of nuclear atypia, cellular pleomorphism, proliferation of fibrous tissue and lipoblasts, as well as the quantification of mitotic activity. Thus, we observed that this score guides the histological diagnosis, helping perform the differential diagnosis (*p* < 0.001). An advantage of this score is represented by the differential diagnosis between all three major entities, not just between lipoma and ALT.

Moreover, we found that this score has a statistically significant value for predicting the results of immunohistochemical and genetic tests of MDM2 and CDK4 (*p* < 0.001) supporting the histopathological diagnosis. These aspects bring an enormous benefit to the healthcare system—the use of as few markers as possible for the same accuracy of the diagnosis—as well as the shorter delivery time of the result compared to the total time of performing the immunohistochemical and genetic tests.

Currently, the gold standard for the diagnosis of lipomatous tumors is the histopathological evaluation. Differential diagnosis is essential due to the clinical evolution, prognosis, and treatment that differ between the different types of adipose tumors [24]. Although the FISH technique has been successfully incorporated into the diagnostic protocol in many academic centers, this modality may exceed the available costs of smaller laboratories [25].

From the point of view of statistical analysis, Asano et al. observed a significant association between the presence of lipoblasts and the diagnosis of ALT/WDLS, as well as between nuclear atypia and this diagnosis, with the specificity of nuclear atypia being 100% for the diagnosis of ALT/WDLS. Because nuclear atypia is absent in benign lipomatous tumors, they are used to exclude a lipoma [21]. In this current research, we observed a significant association between the severity of cellular pleomorphism and the biological potential of the lesions. Thus, severe and moderate pleomorphism correlated with the diagnosis of liposarcoma and low with the diagnosis of ALT/WDLS (*p* < 0.001). Moreover, we identified that a mitotic activity greater than 4.5 mitoses/HPF presents a sensitivity of 82.4% and a specificity of 100% for the diagnosis of liposarcoma.

In the study conducted by Kammerer-Jacquet SF et al., according to the FISH analysis, the 19 ALT/WDLS had MDM2 amplification and 14 had CDK4 co-amplification, and in the benign tumors, the expression of the two markers was negative in 43/44 cases [26]. Thway et al. demonstrated that the p16, CDK4, and MDM2 triad provides sufficient additional information to differentiate ALT/WDLS and dedifferentiated liposarcoma from other adipocyte tumors [27]. In this research, the amplification of the MDM2 gene was present in all cases of ALT/WDLS and 76.47% of liposarcomas, with normal status being preserved in cases of lipoma. Regarding the CDK4 status, we identified the majority of cases with a positive immunoreaction in ALT/WDLS (93.75%), followed by liposarcoma (70.59%) and lipoma (7.59%) (*p* < 0.001).

Thus, FISH testing of MDM2 and CDK4 gene amplifications provides greater accuracy in the diagnosis of ALT/WDLS and is considered the gold standard for differentiating it from a lipoma [21]. In this study, we identified that the MDM2 biomarker is more accurate for the diagnosis of ALT/WDLS than for the diagnosis of liposarcoma. Also, we observed that there is a correlation between the percentage of positive nuclei of the MDM2 and CDK4 markers: CDK4 immunopositivity of over 50% of the nuclei with an MDM2 immunopositivity of over 25% of the nuclei. However, regarding the correspondence between the percentage of positive MDM2 nuclei and the amplified status of this gene detected by CISH, we did not identify a statistically significant association, taking into account the fact that only a percentage greater than 50% corroborated with gene amplification (*p* = 0.282).

The components of the tumor microclimate have an essential role in malignant progression but often in a context-dependent manner. Vascular invasion and the characteristics of blood vessels can be considered to have a prognostic role in certain conditions. In pathological conditions, transient regional and/or chronic hypoxia of the primary tumor is due to dysfunctional vascularization. This characteristic influences the aggressiveness of the tumor and the response to treatment, an aspect observed in the case of sarcomas. In addition, hypoxia contributes to the accentuation of cell migration, invasion, and metastasis [28].

To receive the necessary amount of oxygen and nutrients, so that they can survive and proliferate, tumor cells must be at a certain distance from blood vessels. Hence the need for angiogenesis and the recruitment of its own vasculature [29].

As a tumor grows and reaches the critical size of 1–2 mm^3^, tumor cells distant from blood vessels are deprived of oxygen and nutrients, with tumor growth impaired by apoptosis or necrosis. On the other hand, this phenomenon determines the initiation of angiogenesis to form new blood vessels from the already existing ones [30]. Tumor microvascular density (MVD) assessment is an essential method for quantifying angiogenesis [31].

An important aspect that imposes the need to quantify microvascularization consists in the fact that after the radiotherapeutic treatment, in the cases that lend themselves to this treatment, the MVD does not undergo changes [32].

Cui et al. studied microvasculature in liposarcoma through CD34 and identified an average value of MVD of 56.9/mm^2^, with variations between 12.5 and 225/mm^2^. In their study, MVD was significantly correlated with tumor necrosis, as well as the association with low overall survival. Also, they observed that MVD was an independent risk factor of overall survival [31]. In addition, the study conducted by Virginia Baneth demonstrated that MVD is higher in liposarcomas compared to the other sarcoma included in their study (fibrosarcoma, leiomyosarcoma, and malignant fibrous histiocytoma) [33].

Making a more rigorous analysis that includes two markers (with a role in increasing accuracy), we identified that the highest values of MVD were highlighted in cases of liposarcoma, with an average of 48.98/mm^2^. We observed that a cut-off value ≥38.75 vessels/mm^2^ is highly suggestive of the diagnosis of liposarcoma, with a sensitivity of 94.1% and a specificity of 100%. This cut-off has a higher fidelity in the diagnosis of liposarcomas than the mitotic activity. 

We also observed another crucial role of the microvascular density. When the threshold of 16.25 vessels/mm^2^ is exceeded, an increased presence of nuclear atypias was observed. This threshold can be considered the turning point toward a malignant lesion.

The adaptation of tumor cells to hypoxia is an essential characteristic, key in the development and progression of neoplasia. The role of hypoxia in relation to oncological treatment is to provide resistance to radiotherapy and chemotherapy. It is also associated with a high metastatic potential. In the case of soft tissue sarcomas, hypoxia is responsible for stimulating tumor proliferation and decreasing survival [34]. 

Hypoxia represents the decrease in the internal partial pressure of oxygen in solid tumors below 10–15 mmHg [35]. Tumor hypoxia determines responses, such as the alteration of gene expression, inhibition of apoptosis, stimulation of epithelial-mesenchymal transition, tumor progression, and metastasis but also the stimulation of angiogenesis. Tumor cells adapt to hypoxia by promoting the development of new blood vessels [36]. Hypoxia influences each stage of the neoangiogenic sprouting process. Thus, tumor cell hypoxia represents an important stimulus for the growth of blood vessels [30].

Kim J et al. studied hypoxia in six histological types of sarcomas, including 19 cases of liposarcoma. They observed that the intensity of hypoxia in the case of liposarcomas was lower compared to the other studied categories [37]. In addition, according to the study conducted by Yang L et al., tumor hypoxia was not associated with characteristics such as the age and sex of the patients, the location, and the size of the tumor [38].

Another important element is represented by necrosis. The occurrence of adipose tissue necrosis in benign lipomatous tumors is a rare phenomenon. Most reported cases that included this feature highlighted the presence of necrosis in very large tumors, without a consensus regarding the size threshold [39,40]. A possible cause of this event is ischemia secondary to growth or trauma. Thus, from an imaging point of view, a lipoma with areas of necrosis can imitate an atypical lipomatous tumor [39]. In this present study, we identified the fact that a lesion over 69 mm is more susceptible to the presence of necrosis. 

Therapeutic resources are mainly represented by surgical excision. Often, lipoma is a benign tumor that can be treated conservatively, even if the lesion is large. Surgical treatment is necessary only if the tumor becomes symptomatic. On the other hand, the ALT/WDLS treatment is controversial. If preoperative differentiation from a lipoma is easy to achieve, surgical resection is recommended before the dedifferentiation process develops. The decision on the resection margins is a difficult one due to the high rates of local recurrence. Thus, no significant difference was observed between the marginal and the extensive resection; thus, the conservative surgical intervention of the major blood vessels and nerve structures can be recommended for tumors with deep localization [21]. 

The limitations of this study are represented by the retrospective nature and the absence of imaging data analysis, bearing in mind that not all patients benefited from the same imaging evaluation method, and for some of them, these data were not complete.

The strengths of this work are given by the fact that it is the first study in recent years that emphasizes MVD and identifies its influence in the development of these neoplasms—by identifying some values that play a role in malignant progression (16.25/mm^2^ for the appearance of atypia) as well as in the biological potential (threshold of 38.75/mm^2^ for liposarcoma). Another important element that this study brings is represented by the histopathological score, which meets a group of simple parameters that are easy to follow, which brings a huge benefit to pathologists; not only that, this score has increased predictability and has the advantage of predicting immunohistochemical and genetic results with very high accuracy. Its role helps in improving the differential diagnosis, as well as in the economy of the health system—some centers have difficulties in performing or do not have the necessary equipment for a complete immunohistochemical and genetic panel.

## 5. Conclusions

Adipose tissue tumors represent a broad histopathological spectrum that requires a good differentiation between the common lipoma and the malignant spectrum (ALT/WDLS and liposarcoma). Much research has focused on creating a prediction score, each with its pluses and minuses. In this study, based on the batch and the statistical analysis, we managed to create a histopathological score with increased accuracy, useful both in the differential diagnosis as well as for the prediction of the immunohistochemical and genetic examinations. In addition, the analysis of microvascular density comes with new elements that make it easier to finalize the diagnosis of liposarcoma.

## Figures and Tables

**Table 1:** Identified histopathological types and subtypes.

	Lipoma (*n* = 79)	ALT/WDLS (*n* = 16)		Liposarcoma (*n* = 17)	
**Type**	NOS	Lipoma-like	62.50%	Pleomorphic	17.65%
		Sclerosing	18.75%	Myxoid	35.29%
		Inflammatory	6.25%	Dedifferentiated	47.06%
		Mixed	12.50%		

NOS—no other specified.

**Table 2:** Clinical aspects of the study group.

	Lipoma (*n* = 79)	ALT/WDLS (*n* = 16)	Liposarcoma (*n* = 17)	*p*-Value
Age, years (min–max)	51.81 (3–77)	53.13 (30–68)	61.19 (43–86)	*p* = 0.071
Gender				*p* = 0.489
Female	54.43%	37.50%	52.94%	
Male	45.57%	62.50%	47.06%	
Localization				*p* = 0.161
Head	1.27%	0.00%	5.88%	
Neck	1.27%	18.75%	5.88%	
Upper trunk	30.38%	12.50%	23.54%	
Lower trunk	11.39%	0.00%	0.00%	
Perineal region	8.86%	6.25%	11.76%	
Upper limb	15.19%	18.75%	5.88%	
Lower limb	17.72%	25.00%	29.41%	
Retroperitoneal	13.92%	18.75%	17.65%	
Depth				*p* < 0.001
Deep	100.00%	50.00%	58.82%	
Superficial	0.00%	50.00%	41.18%	
Pain	41.77%	68.75%	76.47%	*p* = 0.010
Maximal diameter, mm (min–max)	63.92 (11–250)	54.75 (13–140)	76 (12–290)	*p* = 0.229

**Table 3:** Morphological and genetic aspects of the study group.

	Lipoma (*n* = 79)	ALT/WDLS (*n* = 16)	Liposarcoma (*n* = 17)	*p*-Value
Nuclear atypia	0.00%	62.50%	100.00%	*p* < 0.001
Karyomegaly	15.19	100.00%	87.50%	*p* < 0.001
Cellular pleomorphism				*p* < 0.001
Low	100.00%	87.50%	29.41%	
Moderate	0.00%	6.25%	29.41%	
High	0.00%	6.25%	41.18%	
Fibrous proliferation	18.99%	81.25%	47.06%	*p* < 0.001
Lipoblasts				*p* < 0.001
Absent	100.00%	0.00%	0.00%	
Rare	0.00%	87.50%	41.18%	
Frequently	0.00%	12.50%	58.82%	
Necrosis				*p* < 0.001
Absent	94.94%	68.75%	29.42%	
<50%	5.06%	31.25%	35.29%	
>50%	0.00%	0.00%	35.29%	
Mitosis/10HPF	0.18 (0–1)	2.19 (1–4)	8.76 (3–19)	*p* < 0.001
MDM2 IHC				*p* < 0.001
Negative	97.47%	12.50%	17.65%	
<25%	2.53%	0.00%	17.65%	
25–50%	0.00%	18.75%	23.52%	
>50%	0.00%	68.75%	41.18%	
CDK4 IHC				*p* < 0.001
Negative	92.41%	6.25%	29.41%	
<25%	7.59%	0.00%	23.53%	
25–50%	0.00%	18.75%	17.65%	
>50%	0.00%	75.00%	29.41%	
Amplification of MDM2	0.00%	100.00%	76.47%	*p* < 0.001
Microvascular density (/mm^2^)	5.50 (3–8.4)	26.33 (24.1–29.7)	48.98 (38.7–84.8)	*p* < 0.001

**Table 4:** Multivariable regression that includes clinical and histopathological data.

Model Summary											
Model	R		R Square			Adjusted R Square			Standard Error of the Estimate		
1	0.906 ^a^		0.821			0.803			0.325		
**Coefficients ^b^**											
**Model**		**Unstandardized** **Coefficients**			**Standardized** **Coefficients**		**t**	**Sig.**		**95.0% Confidence** **Interval for B**	
		**B**		**Standard Error**	**Beta**					**Lower Bound**	**Upper Bound**
(Constant)		1.999		0.463			4.313	0.000		1.079	2.918
Pain		−0.092		0.067	−0.063		−1.384	0.169		−0.225	0.040
Dmax (mm)		−0.001		0.001	−0.078		−1.333	0.185		−0.003	0.001
Depth		−0.021		0.124	−0.010		−0.165	0.869		−0.267	0.226
Nuclear atypia		−0.303		0.142	−0.178		−2.130	0.036		−0.585	−0.021
Karyomegaly		−0.174		0.099	−0.116		−1.750	0.083		−0.370	0.023
Cellular pleomorphism		−0.262		0.091	−0.197		−2.875	0.005		−0.443	−0.081
Fibrous proliferation		−0.298		0.091	−0.191		−3.269	0.001		−0.478	−0.117
Lipoblasts		1.121		0.129	1.036		8.661	0.000		0.864	1.377
Necrosis		−0.074		0.093	−0.055		−0.797	0.427		−0.259	0.111
Mitosis		−0.096		0.020	−0.463		−4.762	0.000		−0.136	−0.056

^a^. Predictors: (Constant), Mitosis, Pain, Dmax (mm), Depth, Fibrous proliferation, Karyomegaly, Cellular pleomorphism, Necrosis, Nuclear atypia, Lipoblasts. ^b^. Dependent Variable: Diagnostic.

**Table 5:** Elements of histopathological score.

Variables	Points
Nuclear atypia	
Absent	0
Present	1
Cellular pleomorphism	
Low	0
Moderate	1
High	2
Fibrous proliferation	
Absent	0
Present	1
Lipoblasts	
Absent	0
Rare	1
Frequent	2
Mitosis	
<2	0
2–4	1
>4	2

**Table 6:** Histopathological score.

Diagnostic	Practical Score	Score	AUC	Sensitivity	Specificity	*p*-Value
Lipoma	0–1	≤1.5	0.997	100.0%	97.0%	<0.001
ALT/WDLS	2–3	≥1.5	0.839	93.8%	82.3%	<0.001
Liposarcoma	4–8	≥3.5	0.980	100.0%	90.5%	<0.001

**Table 7:** The predictability of the histopathological score on MDM2 and CDK4 status.

Marker	Practical Score	Score	AUC	Sensitivity	Specificity	*p*-Value
MDM2 > 50%	≥3	≥2.5	0.915	94.4%	85.1%	<0.001
CDK4 > 50%	≥3	≥2.5	0.879	94.1%	84.2%	<0.001
MDM2 amplification	≥2	≥1.5	0.970	96.6%	95.2%	<0.001

## Data Availability

Data are contained within the article.
